# “I’m a Winner, Not a Victim”: The Facilitating Factors of Post-Traumatic Growth among Women Who Have Suffered Intimate Partner Violence

**DOI:** 10.3390/ijerph19031342

**Published:** 2022-01-25

**Authors:** Hulda S. Bryngeirsdottir, Sigridur Halldorsdottir

**Affiliations:** School of Health Sciences, University of Akureyri, 600 Akureyri, Iceland; sigridur@unak.is

**Keywords:** post-traumatic growth (PTG), intimate partner violence (IPV), gender-based violence (GBV), trauma recovery, healing, rehabilitation, women’s health, phenomenology, qualitative research, interviews

## Abstract

Post-traumatic growth (PTG) is a positive psychological change following trauma. Intimate partner violence (IPV) is one such trauma. The aim of this phenomenological study was to explore PTG from the perspective of women who have survived IPV as well as their perceptions of PTG. Twenty-two female IPV survivors aged 23–56 who reached PTG, according to the working definition used, were interviewed. The overriding theme of the study was “I’m a winner, not a victim”, which describes the essence of the women’s experience of PTG. They described their experience as a shift from being suffering victims of IPV to becoming winners who enjoyed PTG. They felt that their positive attitude and personal strengths had helped them to reach PTG as well as to face the fact that they had been in an abusive relationship, thus forgiving and believing in themselves and taking responsibility for their own health and well-being. They sought knowledge about violence, how to process it, and how to respond to triggers. They set boundaries for their perpetrators and were in as little contact with them as possible. They chose the company of positive, supportive, and constructive people and situations where they were not being controlled. It was concluded that, even though suffering IPV is a terrible experience that no one should endure, the participants’ experiences had resulted in PTG that they treasured.

## 1. Introduction

When confronting the entire range of debilitating effects of trauma, most survivors display a stunning capacity for survival and persistence [1,2]. Leaving an abusive relationship means great changes in life for a survivor of IPV. Some survivors shift from survival mode to starting a new life, going from being controlled to being in control of their own lives [3]. Research on IPV has mostly been focusing on the negative consequences of that experience. Even though that part of their experience should not be minimized, their strength, resilience and other positive resources of their recovery should be acknowledged and emphasized. Women can recover from experiencing IPV, but there is lack of information on how they recover and if their recovery is long-term [1,4].

Gender-based violence (GBV) is a widespread and serious societal problem. Nearly one in three women around the world is affected by GBV, regardless of their social circumstances or ethnicity. The most common type of GBV is domestic violence (30%) [5]. In the UN Declaration on the Elimination of Violence against Women, GBV is defined as “any act of gender-based violence that results in, or is likely to result in, physical, sexual or psychological harm or suffering to women, including threats of such acts, coercion or arbitrary deprivation of liberty, whether occurring in public or in private life” [6,7]. Victims of GBV are at increased risk of depression, having thoughts of suicide and attempting suicide, physical injury, symptoms of mental disorder, unwanted pregnancy, the HIV virus, various diseases, and being killed by a spouse [8]. The experience of gender-based violence is also linked to post-traumatic stress disorder (PTSD) [1,9,10], and many GBV victims meet criteria for a range of mental and physical illnesses and suffer medically unexplained symptoms [1,11,12,13].

Intimate partner violence (IPV) is one of the most common forms of violence against women. IPV is any behavior in an intimate relationship that causes physical, psychological, or sexual harm to the victim of the violence. IPV also applies to controlling behaviors such as isolating the partner from other people, monitoring their doings, and controlling and restricting finances, employment, education, and medical care [14,15]. International research has shown that a woman is more likely to be assaulted, injured, raped, or killed by a current or former partner than by any other person, and the perpetrator is most often a male [9,16]. Psychological aggression is the most common form of IPV and is an even stronger predictor of PTSD than physical violence. Psychological violence is likely to reduce self-kindness and is thus related to less meaning in life, less positive reframing of stressful events, and result in less growth and maturity in the victim of IPV [17]. Victims of IPV also often lose their internal ego structure [18]. Leaving an IPV relationship is a complex process that takes place over time, even after the violent relationship has ended [4]. The post-IPV trauma effects include negative physical and mental outcomes and also negative financial consequences, housing instability, and social stigma [19]. It takes tremendous strength to shift from a survival mode to starting a new life after putting an end to an IPV relationship [1].

Post-traumatic growth (PTG) is a positive psychological change by a person following severe difficulties and trauma. In PTG, the potential for positive outcomes associated with trauma is explored rather than focusing on the negative consequences [20]. In PTG, the person experiences increased spiritual maturity, discovers new opportunities in life, values life more, experiences increased personal strength, and has better relationships with others [21,22]. These individuals also discover new possibilities in life and experience positive psychological changes in themselves [23,24,25]. In assessing PTG, all these factors are considered [25]. In a qualitative study of PTG, where 12 men and women with different backgrounds, history, and trauma experience participated, PTG was described as a journey rather than a destination. Participants described how their personal factors and their need for change inspired their journey toward PTG [26]. The results from that research indicated that more attention should be paid to people suffering psychological trauma so that serious long-term negative consequences can be minimized or even prevented. However, we need to better understand the factors facilitating PTG among groups of people with defined trauma suffering.

### Purpose of the Study and Research Question

This study is part of a larger research project aimed at developing a theory on post-traumatic growth among women who have suffered intimate partner violence. The aim of the present study was twofold: (1) to explore the factors that facilitate PTG from the perspective of women who have made the shift from suffering victims of IPV to survivors who enjoy PTG and (2) to learn about participants’ perceptions of PTG. The main research question was: What are the facilitating factors of Post-Traumatic Growth among women who have suffered Intimate Partner Violence and what is their perception of Post-Traumatic Growth?

## 2. Materials and Methods

### 2.1. Study Design

**Figure 1:**
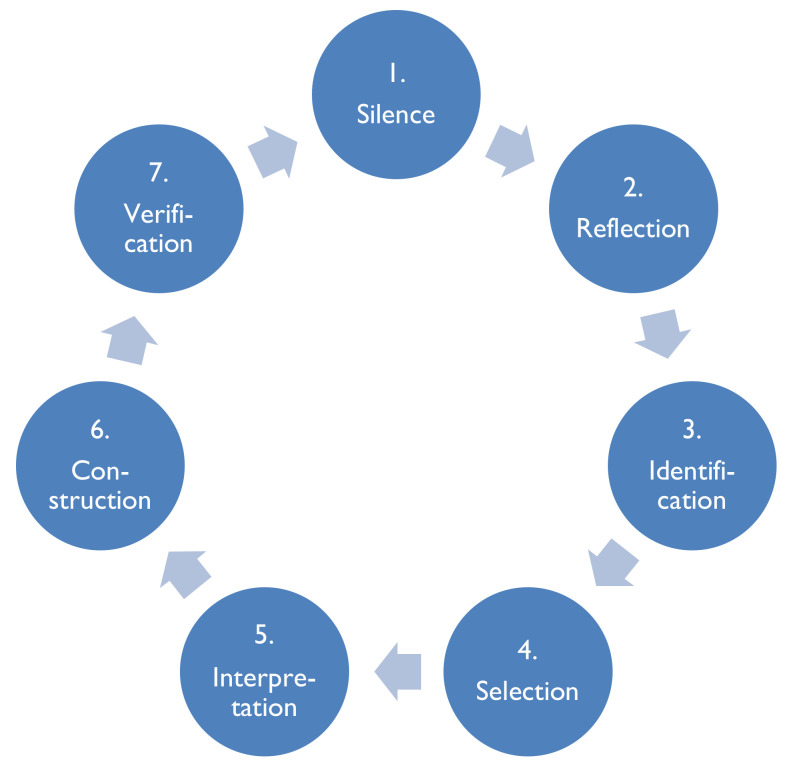
The research process of doing phenomenology from the Vancouver School ([31], p. 56). Used with permission. This cycle is repeated in every one of the 12 steps of the Vancouver School process.

The research methodology chosen to answer the research question was from the Vancouver School of Doing Phenomenology (The Vancouver School), which traces its roots to the works of Spiegelberg [27] in a unique blend with Ricoeur’s [28,29] hermeneutic phenomenology and Schwandt’s [30] constructivism. The Vancouver School aims to understand the experience of participants in a particular phenomenon. Participants describe their experience of the phenomenon, and the researchers aim to understand and describe their experience. The purpose of this method is to improve human services through increasing the knowledge and understanding of human phenomena [31]. An overview of each of the steps in the Vancouver School research process is found in Figure 1.

### 2.2. Participants

A purposeful sampling was used. Participation was voluntary and anonymous. The inclusion criteria were having reached PTG (see working definition used in Table 1), being 18 years or older, and being able to read and understand Icelandic, with at least one year having passed since the end of the violent relationship. A total of 34 women signed up for interviews on PTG, but then the COVID pandemic commenced. When interviewing became possible again, 22 women in the age range of 23–56 years old (M = 40.5) were eventually interviewed.

### 2.3. Data Collection and Analysis

The 12 basic research steps of the Vancouver School process were followed in the data collection, and the data analysis followed in each of the steps (see Table 2). An interview schedule developed by the researchers based on the research question and the literature was followed, and yet participants were encouraged to express themselves freely and openly. The interviews, ranging from 39 to 134 min (M = 77 min), were recorded and encrypted. The first author conducted all the interviews and did the preliminary data analysis under the supervision of the second author.

### 2.4. Research Ethics

The main principles of research ethics guided researchers in the study. The National Bioethics Committee granted permission to conduct the study (reference no: VSN-19-166). Each participant received an introductory letter and signed an informed consent before the beginning of the interview. In the letter, potential participants were informed about the purpose of the study, the research method, and what was involved in participation. They were informed of their rights to participate voluntarily and to withdraw from the study whenever they so wished, as well as of their anonymity and the absolute confidentiality. Participants were offered professional psychological support free of charge if they felt the need. No participant took advantage of such support.

## 3. Results

**Figure 2:**
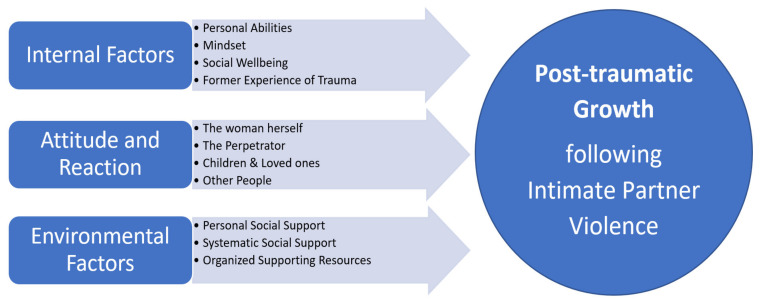
Facilitating factors in post-traumatic growth following intimate partner violence.

The overriding theme of the study was “I’m a winner, not a victim”, which described the women’s essential experience of PTG. They described their experience as a shift from being suffering victims of IPV to becoming winners who enjoyed PTG. They felt that their positive attitude and personal strengths had helped them to reach PTG, as well as to face the fact that they had been in an abusive relationship, thus forgiving and believing in themselves and taking responsibility for their own health and well-being. They sought knowledge about violence, how to process it, and how to respond to triggers. They set boundaries for their perpetrators and were in as little contact with them as possible. They chose the company of positive, supportive, and constructive people and situations where they were not being controlled. All participants had suffered emotional and psychological IPV as well as verbal abuse and oppression. Many of them also described physical and sexual violence as well as financial abuse. The women had all undergone the shift from suffering victims of IPV to winners who enjoyed PTG, and they described the factors that facilitated their PTG. They saw the journey to PTG as an ongoing process, where certain factors facilitated their PTG. The findings of these facilitating factors among women who suffered IPV are presented in Figure 2.

The three main facilitating factors in participants’ PTG were (1) internal factors: personal abilities and mindset, social well-being, and former experience of trauma; (2) attitude and reaction to herself, the perpetrator, her children, her loved ones, and other people; and (3) environmental factors: social support and organized supporting resources.

### 3.1. “I Decided Not to Be a Victim”: Internal Factors Facilitating PTG Following IPV

Participants explained how their internal factors facilitated their PTG following their experience of IPV. They expressed how possessing positive personal abilities, facing the situation, and being determined in improving their situation, facilitated their PTG. They also described the necessity of meeting their own needs for well-being, so they could focus on their PTG. Most participants considered the experience of dealing with and processing former traumatic events helpful in their PTG and described how that experience had served as some kind of preparation for their traumatic experience later in life.

**Personal abilities**. The women stated that the congenital, inner personal abilities, along with their personal skills they had developed through life, had been helpful when processing their experience of IPV and finding their way to PTG. Their positive attitude and personal factors turned out to be helpful on that journey to their current situation in life. They described the importance of possessing resilience, where they did not give up despite adversity and continued their journey to PTG, together with personal strengths such as the will to fight, stubbornness, and positivity.

“*I think it’s my resilience, I’m incredibly strong, even though many people have tried to break me, no one has succeeded*”

(Norma).

“*I can deal with things that are incredibly difficult and I don’t bend, and I stand on my own two feet… I have incredible faith in myself*”

(Ingrid).

**Mindset.** The participants reported that their own attitude and opinion about their situation mattered. They described the importance of themselves realizing and facing the fact that they had been in an abusive relationship.

“*Being able to say it out loud that I have been traumatized and I have suffered mental abuse… I had a very hard time admitting that to myself*”

(Kathy).

They described the importance of making the decision themselves to seek help, to stand by themselves, to believe in themselves, and to take responsibility for their own health.

“*In the end, all the help in the world could not have helped me if I didn’t want it… I had no plans of being a victim*”

(Harriet).

“*As long as you reach out and say, “I need help”. We are all different and I need something and someone else needs different things… don’t ignore that you need to rebuild yourself, both mentally and physically*”

(Audrey).

It was important for them to work on forgiving themselves, stop blaming themselves, and returning the shame.

“*I had to understand myself completely. Understand why I made the wrong decisions over the years. I returned the responsibility of the violence and the shame too*”

(Audrey).

The women did not want to feel sorry for themselves or to be victims. They wanted to be fearless, have faith in their own abilities, be happy, look toward the future, and move on with their lives.

“*I just wasn’t going to become some crumb of bread laying on the earth… I was not going to let him defeat me forever like my grandma did. To be a prisoner of a situation that you live with throughout your life*”

(Vera).

“*I was going to be happy… for me and my kids, the goal was to be healthy in all fields*”

(Thelma).

**Social well-being.** Participants described that meeting their basic needs in life, such as safe living conditions, safe housing, and financial independence, following the IPV led to a certain stability and gave them the opportunity to focus on their journey to PTG.

“*I was able to move immediately in with my mother where it was safe for me and my child so I didn’t have to worry about where I lived or that I would be homeless… I could be calm. I had my safety net and secure income*”

(Joan).

“*I took a loan to fix things in my life and I assumed half a year without income when I took the loan… I only gave myself half a year, which turned out to be 9 months*”

(Audrey).

**Former experience of trauma.** Most participants had former experience of trauma or adversity in their childhood, prior to their endurance of IPV. Many women described how their previous experience of trauma and challenging life experiences had strengthened their interpersonal skills, serving as a certain preparation for the traumatic, violent relationship as well as for PTG.

“*This is the mode, the reaction to the violence I experienced as a child, I’m a survivor and not a victim, it also helped me in this. I went into the same mode there… survivor of domestic violence*”

(Thelma).

“*I think it’s also a kind of survival mode I fall into. I think it’s something I learned when I was younger to be so hard and cold and I seem to be able to turn off emotions a lot, unfortunately. It’s both an advantage and a disadvantage but I think it helped me a lot*”

(Paula).

The main facilitating internal factors in participants’ PTG are explained in Table 3.

### 3.2. “It Doesn’t Always Have to Be This Way”: Attitude and Reactions to Self and Others Facilitating PTG Following IPV

All participants talked about how they consciously worked on making themselves stronger and described how they responded to themselves, the perpetrator, their loved ones, their children, and other people. The women decided themselves that they were going to have a better life and a life on their own terms. They chose carefully where to seek help and consciously took the time they needed to process their experience and feelings, being kind towards themselves while doing so. They appreciated their loved ones more and consciously nurtured their relations to those people. The women set reasonable goals, chose the company of constructive people, and took control of their lives, always aiming for a brighter future on their own terms.

**The woman herself.** Participants consciously took action to promote their own recovery and PTG in the aftermath of IPV. They emphasized the importance of sharing and discussing their experience and feelings.

“*It was really just one, two, three, go, to start talking about this and look at this from a different perspective rather than from the inside of the turbulence somehow*”

(Fanny).

They described the importance of processing their experiences and cultivating their mental and physical health.

“*I want this core, that I am, to shine, so I just started taking stuff from my backpack, relieving it, empowering myself, doing what I think is awesome. On my own terms*”

(Ingrid).

The women accepted their feelings and allowed themselves to experience both bad and good feelings, being understanding and caring toward themselves. That way, they came to know themselves again. Many of them made changes in their lives, e.g., started to study, moved to new areas, and found new hobbies.

“*We should allow ourselves to go into a time of sorrow and when you’re in difficult periods in life it is natural that you don’t feel well, but then you have to find a way out of it and know that there is a way out of it. It doesn’t always have to be this way. That’s very important*”

(Eve).

“*I had lost myself in this role of being a wife and mother and I no longer knew who I was. I, the character Georgia, I started trying to find myself again and that was my huge task.*”

(Georgia).

The women took control of their lives, looked to the future, and set realistic goals for themselves. They worked on their co-dependence and learned how to set boundaries. They returned their sense of shame for having been abused, took the time they needed to process their experience, and built up their growth in a way that suited them.

“*Focusing on me. Cultivating the body and the soul. Talking about my experience and just fighting. Relaxing and fooling around“*

(Ingrid).

“*The focus has become very clear, how I want things to be and what I accept and what I don’t… Now I set goals and if people can’t respect me, they can be somewhere else*”

(Norma).

They found it important to gain knowledge about violence and about how to process the experiences of violence, as well as to learn to recognize and respond to what triggered them.

“*I’ve realized what triggers me. It feels good to have learned to know these triggers, how to deal with them, to give myself time to stop and just think, okay this is it, to understand why I am feeling this way. Instead of just suppressing my feelings and then just explode one day*”

(Ursula).

**The perpetrator.** The women set boundaries for their perpetrators and were in as little contact with them as possible. They decided not to let them control their lives and took that control into their own hands.

“*To not care about what he thinks of me… no matter what he’s saying about me because you know people do not take him seriously because he’s just crazy or he’s just sick… of course there were difficult times where you might get some distressing emails or… [need] to communicate with him… but it was so easy for me not to be with him anymore*”

(Rose).

“*The only way he is allowed to communicate with me is through e-mail and during one period we were considering that he could just send my brother an e-mail. Just so I wouldn’t get distressed when receiving these e-mails. There were all kinds of rules that we set up just so I could have space and peace to recover*”

(Joan).

**Children and loved ones.** Most participants were mothers. Many of them felt bad because of what their children had suffered because of the IPV. They asked their children for forgiveness and forgave others.

“*I can ask them to forgive me, for the mistakes I made, and I know they do. I know they don’t judge me. We all make mistakes but sometimes the mistakes are too serious and cost too much*”

(Eve).

Most women described how their children were their inspiration in their PTG following the violent relationship.

“*The love for my children is what kept me alive… I think I wouldn’t have survived without the responsibility for them… I want to be there for them, to be a role model*”

(Lorna).

They valued their loved ones and their friends more than before and took better care of their supporting network, but they also set boundaries for those people to protect their own independence and remain in control of their own lives.

“*It is extremely important in the recovery process to have people around you who love and respect you, help you… you need support*”

(Eve).

“*You must be trained in setting boundaries and you have to be trained in maintaining your own hearing or in listening to yourself*”

(Joan).

**Other people.** The women said they chose the company of positive, supportive, and constructive people and situations where they were not being controlled. They sought help in places that felt right for them. They tried not to take things personally and were very much aware of their well-being and feelings when communicating with the opposite sex.

“*I had and I still have very respectful friends. They were not saying “you have to do this”. My friends just sat there, “do you want a hug”, “shall we talk”? There was never any “you must”. I just got to be me, being in control, and if I was making the wrong turn someone just guided me gently in the right direction… I was really lucky*”

(Paula).

“*It was important to me to learn how to seek support in places where I knew that I would be supported*”

(Ursula).

The main facilitating factors of attitude and reaction in participants’ PTG are explained in Table 4.

### 3.3. “To Get This Opportunity… ”: Environmental Factors Facilitating PTG Following IPV

The women talked about the importance of a supportive environment when building a better life, on their own terms, and processing PTG following IPV. To communicate with supportive and warm people whom they trusted, and to get the help they needed, encouraged them in making positive changes in their lives, thus enhancing their PTG. They emphasized the importance of their informal support (e.g., friends, family, coworkers), systematic support (e.g., the public system), and organized resources providing formal support, which they used to deal with the aftermath of IPV and to work their way to PTG (e.g., the Women’s Shelter, and other resources for women who suffer IPV).

“*I consulted in friends and relatives, especially friends to talk and talk, to relatives for help with the children. I turned to the social services for financial support so I could provide more for my children, enabling them to practice sports beside the sports at school. I turned to charity agencies for food, I also looked for all possible ways in the system to get help*”

(Anna).

**Informal support.** The participants described the importance of experiencing various support from their family and friends, new spouses, colleagues, neighbors, other people, and even their pets, when working on their PTG.

“*To feel that my people would love me even though I went through all this. I was always afraid that people would look at me differently, that people would see me as a victim or think: “what was she thinking, she can blame herself, should I feel sorry for her?” I have never received such a response from anyone*”

(Fanny).

“*My current husband has been very helpful, he has helped me to find purpose in life and to enjoy life*”

(Urma).

“*To come home after work… and there she is, so happy to see me, just “hi mom where have you been? Can we go outside?”… my dog, I put all my trust in her and she really got me through this. Because she was always there for me… We were very close*”

(Ingrid).

**Systematic support.** The women described various useful public resources they found helpful when working on their PTG. Some women found the public system very useful and even described groundbreaking moments when communicating with professionals in the system, such as psychologists, doctors, nurses, physical therapists, social workers, social services, family therapists, police, priests, lawyers, as well as various professionals in rehabilitation resources and social services.

“*I got a lot of support from the police… they drove past my workplace and my home; you saw the police on the move*”

(Eve).

“*Finally, I went to my physician and told him everything… and of course he saw how I felt. That’s how it all started… he prescribed antidepressants and applied for rehabilitation program for me… I was really lucky to meet such an understanding physician*”

(Lorna).

**Organized supporting resources.** Even though the support from their loved ones, friends, and the system was helpful, it was important to obtain help from people who knew IPV or were specialists in that field. Participants also described several organized supporting resources that had been useful for them when processing PTG. Often those resources worked together with or without the public system when helping the women.

“*It’s not always enough to talk to a friend or a relative, because there is so much violence in families that no one is aware of. Talk to someone who knows violence. When I told my mum that I was getting a divorce because my husband was violent to me, she just said: “But what about the house?”* (laughter)”

(Ursula).

“*The staff at the Women’s Shelter helped me go to this trauma team at the hospital… I went there for interviews... for a year and a half. It also helped me, strengthened me a lot*”

(Mona).

“*Attending this rehabilitation program and meeting with this consultant supported me a lot… I went to all kinds of interviews... I met a psychologist, went to physical therapy, to the gym… I was just finding myself back there*”

(Lorna).

The main facilitating environmental factors in participants’ PTG are explained in Table 5.

### 3.4. “Now I Can Get through Anything…”: Participants’ Perceptions of PTG Following IPV

All participants described their perceptions of PTG following IPV. The women identified 16 descriptive concepts when describing their perceptions of PTG following IPV. The concepts identified were cited by at least half of the participants of this research.

Participants agreed that, although suffering IPV was a terrible experience, which no one should have to endure, their experience had resulted in positive outcomes in many ways. An overview of the women’s perceptions of PTG following IPV are presented in Table 6.

**Strength.** Participants emphasized their enormous personal strength. They described how their personal strength had grown, giving them the feeling that they could overcome all difficulties in life. They said they were not afraid anymore.

“*I can do so much more than I thought I could. If you are willing to work on your life, even though it takes time and can be really scary and tough… at the end you will get what you worked for*”

(Kathy).

“*I just know, that after this experience I can get through anything*”

(Harriet).

**Self-Respect.** The women got to know themselves better than before, accepting the person they are today. They said they respected themselves and enjoyed their own company. They were proud of what they had accomplished in life.

“*I really enjoy my own company and my soul is peaceful most of the time. I have learned how important it is to take good care of myself. I’m really proud of myself*”

(Ursula).

**Appreciation.** Participants described how they had got to know themselves and appreciated themselves more than before. They said they accepted themselves as they are.

“*At last I love my self… I think I deserve all the best*”

(Norma).

**Boundaries.** Participants were aware that they were not responsible for what other people do, say, or think. The women said that they were also aware of what they wanted in their lives and what they did not want, which is why they consciously set boundaries toward other people and themselves.

“*It has become clear to me, how I want things to be, what I accept from other people and what I do not accept*”

(Norma).

“*You just learn so much about yourself. You deal with it when other people treat you badly and you set boundaries towards them. You listen to yourself and have to learn when you must set boundaries for yourself… it’s okay to be yourself, to have normal communication with others and yourself*”

(Joan).

**Tolerance.** The women described how they had become more tolerant toward other people. They said that they were humbler towards others, they showed more kindness, and were more patient and understanding of other people.

“*I have learned to be humble towards other people and to respect their experience*”

(Harriet).

**Awareness.** Participants described how they had learned to know themselves, realizing and accepting their own needs. They said that they took better care of themselves than before.

“*I have learned so much about myself. Who I am, how I feel, what I want, what I need and so on. I’ve also learned what I don’t want in my life*”

(Ursula).

**Independence.** The women said that they feel more independent and braver. They can do what they want to do without getting permission from someone else.

“*I can make a big decision and make it happen. I don’t need permission from anyone. I can do everything I want to, I’m an independent and good person*”

(Beth).

**Selfhood.** Participants said they felt whole. They emphasized that their difficult experience of IPV had made them the people they are today.

“*This experience made me the woman that I am today… I could be drinking and whining every day, but I choose not to. I figured things out instead and became so strong and happy… I became me*”

(Paula).

**Happiness.** Despite their difficult experience of violence, the women said that most days they feel good, are happy, and enjoy life.

“*I’m so happy, I feel really good. Even though I feel sad some days… I know the best way for me to handle those feelings and work on my welfare*”

(Mona).

“*Life is so much more beautiful and better since I got out of this [violent relationship]… I really appreciate myself… some days I fake it till I make it, I admit that, but most days life is so much better for me*”

(Mona).

**Nurture.** Participants took better care of themselves than before. They loved themselves and worked on their own happiness.

“*I know I must take good care of myself and I do. I have this routine, that I need to feel good, the more I take care of myself, the better I feel*”.

(Lorna).

**Vision.** The women saw their future as bright and joyful and felt that they were in control of their lives.

“*My future is maybe unclear, but it’s bright. Now I have the strength to build a good life for myself and my children*”

(Thelma).

“*The future... there is so much I can do… I really look forward to it*”

(Olivia).

**Helpfulness.** Many participants described their need for using their experience of IPV to help other people suffering violence. That was, in many cases, the reason for their participation in the study.

“*I really want to use my experience to help other people who have suffered violence… that’s why I am participating in this study*”

(Rose).

**Resilience.** Participants described their experience of IPV as a difficult experience. However, many of them stated that they would not want to be without that part of their lives, since that experience had resulted in so many positive things for them.

“*To be in a violent relationship is a terrible experience. Even so, the growth that followed is so enormous, so precious… I wouldn’t want to be without that growth*”

(Joan).

**Empowerment.** The women described how they had grown to feel in control of their own lives.

“*I have learned so much. I feel so empowered. I’m in control of my life… that is the things that are controllable. I know I will get through everything. I will never give up*”

(Ingrid).

“*I’m whole now. It’s like the pieces of me have been glued together again. I’m responsible for my actions, my needs, my well-being and what I do to preserve my well-being. Other people are responsible for their well-being and actions, that’s not my responsibility*”

(Norma).

**Reinforcement.** Participants were aware of and responsible for their own well-being. They emphasized the importance of seeking help when they needed it in places that they felt were right for them.

“*To seek help when you need it… in places where you know you will get the help and support that you need… that was a very important lesson to learn*”

(Ursula).

**Determination.** When it came to current and/or future intimate relationships, the women knew what they wanted. They were determined to focus on their needs and their desires in their new or future intimate relationships. In general, the women were ready to fight for justice.

“*If I will find another partner in the future one thing is clear: I’m not going to fix you, I’m not going to be your mother, I want a spouse that is my equal*”

(Georgia).

**Figure 3:**
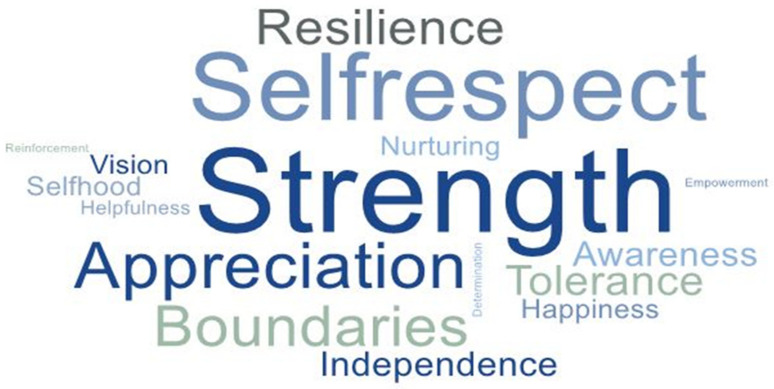
Participants’ perception of PTG following IPV.

The main concepts that at least half of the participants used to describe their perception of PTG following IPV are portrayed in Figure 3. The size of the concepts shown in this picture depends on how many participants reported it as a part of their PTG, the most common descriptive concept being the largest one.

Ten of the descriptive concepts of PTG were intrapersonal, where the women described how they saw themselves and their personal feelings toward themselves in a more positive way (strength, self-respect, appreciation, awareness, independence, selfhood, happiness, nurture, empowerment, and reinforcement). Two concepts were interpersonal and reflected their changed behavior and feelings toward others (tolerance and helpfulness). Four concepts were both intrapersonal and interpersonal, where their self-reflection possibly included and affected other people (boundaries, vision, resilience, and determination).

## 4. Discussion

This article is a valuable contribution to the field of research on IPV and PTG. The results reveal that, in spite of serious and often long-term consequences of IPV, participants succeeded in having good lives and enjoying PTG. It is important for both victims of IPV and people supporting them to be aware of the possibility of PTG, as well as the factors facilitating that growth. To be aware of this fact can in that way bring hope to survivors of IPV, their loved ones, and professionals working in the field of IPV. This article is also an important contribution to the field of PTG, since the results reveal that the expression of PTG, as well as the facilitating factors in PTG, can differ, depending on types of trauma. 

While trauma effects following IPV are well-known, researchers have paid less attention to the facilitating factors of healing from trauma and the possibility of PTG for survivors of IPV, although such literature is increasing. Healing has been shown to be comprised of connecting with the self, others, and the world [32], while PTG seems to also involve that the person experiences increased spiritual maturity, discovers new opportunities in life, values life more, experiences increased personal strength, and has better relationships with others [21]. Since IPV is of an intimate, complex, and chronic nature, the trauma recovery is unique [19], and some researchers have described it as unlikely and even undesired [33]. The purpose of this study was dual: to identify the factors facilitating PTG among female survivors of IPV and their perception of PTG following IPV. The results of this study were based on the descriptions of 22 female participants in the phenomena. The overriding theme of the study was “I’m a winner, not a victim”. Those words describe the core of the participant’s experience of PTG, where they shifted from being suffering victims of IPV to becoming winners who enjoyed PTG. The participants in the study were determined not to let their experience of violence affect their whole lives in a negative way. They did what they had to do to take the control of their lives and consciously promoted their recovery and PTG in the aftermath of IPV. They described how their positive attitude and personal strengths had been helpful in reaching PTG and confronted the fact that they had been in an abusive relationship. Many participants reported safe living conditions and support as important factors in PTG, asserting that being free of those worries gave them the peace they needed to work on their growth. Similarly, the importance of safe housing along with addressing concerns such as safety and trauma-related issues were important factors for long-term success in processing IPV, as was reported in another study [34]. Many of the women reported that their previous experiences of trauma and difficult life experiences had often been helpful when dealing with their circumstances, thus contributing to their PTG. Similarly, in a qualitative study of PTG among people with a history of various types of trauma, participants described how their earlier traumatic experiences turned out to be helpful in dealing with their traumatic experience later in life and reported this as a facilitating factor in their PTG [26]. Participants consciously worked on their attitudes and reactions to themselves and others. They forgave themselves and others, believed in themselves, and took responsibility for their own health and well-being. They sought information about violence, how to process it, and how to realize and respond to triggers related to their experience of IPV. Moreover, the women set boundaries for other people, which resulted in better relations with the people they wanted in their lives and fewer interactions with their perpetrators. In a similar way, a qualitative study on themes of healing and PTG in female survivors of IPV described how participants sustained and created new limits on others, resulting in improved relationships with their loved ones [35]. Participants in the present study described the importance of a supportive environment and chose the company of positive, supportive, and constructive people and situations where they were not being controlled. Likewise, in a literature review of informal social support for survivors of IPV, a connection was found between positive social reaction and psychological health benefits and fewer negative health symptoms [36], and in a study on the role of social support for PTG among female victims of IPV, the results indicated that social support predicts higher levels of PTG [37]. The women discussed the importance of getting different kinds of systematic support from the public system and organized resources for working their way to PTG. They emphasized the importance of receiving the right kind of support from different organized resources on their own terms.

The participants in this research described their perception of PTG following IPV, where 16 descriptive concepts of PTG were identified. These descriptive concepts of PTG reflected that the women saw themselves, their lives, and their future from a new and more positive perspective. They had also discovered that they had their rights in life and were allowed to be happy, like everybody else, so they did what they had to do to take the control of their lives. They chose their company carefully and set boundaries, which led to better relationships with other people. Even though several studies have shown that previous traumatic experiences could be associated with revictimization in IPV [38,39], that was not the case by the women in this study. When discussing current and/or future intimate relationships, they sounded determined to focus on what they want and what they need in that regard. Participants agreed that even though their experience of IPV had been difficult, it was valuable to them, since it had made them the people they are today. It was an interesting outcome of this study that the majority of the concepts used by the women to describe their PTG were intrapersonal (strength, self-respect, appreciation, awareness, independence, selfhood, happiness, nurture, vision, resilience, empowerment, and determination) or both intrapersonal and interpersonal (boundaries and reinforcement), when only two concepts describing PTG were interpersonal (tolerance and helpfulness). This outcome suggested that women who have suffered IPV perceive PTG to a large degree as a personal, inner growth and reconstruction of themselves. This was a different focus from the working definition of this study (see Table 1) where the factors constructing PTG seem to have equivalent relevancy when describing PTG.

The results of this study emphasized the complexity of PTG among survivors of IPV and the necessity that all these factors be considered in a holistic way when supporting survivors of IPV on their journey to PTG. Equally important findings of this study were the participants’ descriptions of their perceptions of PTG following IPV, the main focus being on their own intrapersonal factors.

### Limitations of the Study

Participants self-reported their PTG, and therefore, the sample selection may involve a bias in that regard. The participants’ willingness and ability to express themselves regarding their experience of the factors facilitating PTG and their perception of PTG following IPV could be a limitation to this study. The authors do not have information about the participants’ socioeconomic status, religion, family status, or occupation, which could be a limitation of the study. It could also be a limitation that one of the criteria for participation in this study was being able to read and understand Icelandic, which may have led to bias due to possible homogeneity in the culture of the sample. 

## 5. Conclusions

The current study illustrates findings that provide a deeper understanding of the journey to PTG following IPV. The results suggest that encouraging and assisting survivors of IPV to systematically work on specific facilitators not only motivates them but also results in their PTG. This information can be useful when guiding and supporting women who have suffered IPV to start and/or continue their journey toward PTG. Furthermore, the results indicate that the same definition of PTG does not apply to all groups of people. The results reveal that participants of this study, who all enjoyed PTG following their experience of IPV, were well-aware of what they wanted from their current and/or future intimate relationships, which suggests diminished danger of them being revictimized. It could be useful to do a future research on that subject, in order to reveal the possibility of PTG being a protective factor in revictimization of women who have suffered IPV. In this study, the women described their perceptions of PTG and the factors facilitating PTG. A description of the factors hindering PTG among these women would create a clearer picture of the development of PTG in this group. Thus, it would be useful to perform further research on the factors hindering PTG among women who have survived IPV.

## Figures and Tables

**Table 1:** The working definition of post-traumatic growth (PTG).

An individual who has reached post-traumatic growth experiences positive personal changes as a result of a struggle with a traumatic event. The individual has increased personal strength, improved relationships with others, experiences positive changes in attitudes and appreciation towards life, and sees new possibilities in life. The experience, though negative, has had positive meaning for the person.

The researchers based their working definition of PTG on their own definition already published ([26] p. 4).

**Table 2:** The 12 basic research steps of the Vancouver School process and how they were followed in the present study.

Steps in the Research Process	What Was Done in the Present Study
Step 1. Selecting dialogue partners (*the sample*).	Thirty-four women who believed they fulfilled the working definition of PTG signed up for interviews on PTG, but in the meantime the COVID pandemic began. When interviewing became possible again, 22 women still wanted to participate and were interviewed.
Step 2. Silence (*before entering a dialogue*).	The researchers reflected upon their preconceived ideas and consciously put them aside as much as possible.
Step 3. Participating in a dialogue (*data collection*).	One interview was conducted with each participant. The first author conducted all the interviews, which were recorded, transcribed verbatim on a computer, and encrypted.
Step 4. Sharpened awareness of words (*data analysis*).	The interviews were read multiple times by the first author. Comments were written in the margins to find the core of the interview and to answer the research questions. Nvivo12 was also used.
Step 5. Beginning consideration of essences (*coding*).	Each interview was analyzed in detail, and main themes and subthemes were constructed.
Step 6. Constructing the essential structure of the phenomenon from each case (*individual case construction*).	The main themes and subthemes of each participant’s story were highlighted, and the most important themes constructed into an individual analytic framework.
Step 7. Verifying each case construction with the relevant participant (*verification 1*).	Many participants were very emotional during the interview. Because of the delicacy of the subject participants were not asked to confirm their individual analytic framework.
Step 8. Constructing the essential structure of the phenomenon from all the cases (*meta-synthesis of all the different case constructions*).	All individual analytic frameworks were compared and constructed into one main analytic framework.
Step 9. Comparing the essential structure of the phenomenon with the data for verification (*verification 2*)	For verification, all the transcripts were read over again and compared to the final analytic framework.
Step 10. Identifying the overriding theme describing the phenomenon (*construction of the main theme*).	“I’m a Winner, not a Victim”: The Facilitating Factors in Post-Traumatic Growth among Women who have suffered Intimate Partner Violence.
Step 11. Verifying the essential structure with the research participants (*verification 3*).	Many participants were very emotional during the interview. Because of the delicacy of the subject participants were not asked to confirm the final analytic framework or the main theme.
Step 12. Writing up the findings (*multivoiced reconstruction*).	The participants were quoted directly to increase the trustworthiness of the findings and conclusions.

**Table 3:** Internal factors facilitating PTG following IPV.

Internal Factors	
Personal Abilities	Possessing resilience/strength, the will to fight, stubbornness and ambition. Being positive, optimistic, calm, and peaceful (serenity). Having courage, self-confidence, self-esteem. Consciousness, being organized, possessing social skills, being quick to forgive and let go.
Mindset	Being ready to: face the situation, get help, take responsibility for herself, forgive herself, care for herself. Possessing the will to feel good, finding her own strength, and having faith in better life to come. Not being a victim.
Social Circumstances	Finding a safe place to stay and/or live, financial security.
Former Experience of Trauma	Experiencing adversity in childhood, former processing of trauma, standing strong before the violent relationship.

**Table 4:** Attitude and reactions to self and others facilitating PTG following IPV.

Attitude and Reactions to Self and Others	
The Woman Herself	Taking good care of herself; working on mental and physical health, showing herself compassion, allowing herself to be emotional, taking the time needed to process the experience of trauma, heal, and settle with her experience. Taking control in her life; getting to know herself again, finding the purpose in life, setting reasonable goals and looking toward the future. Not taking responsibility for the violence; returning the shame to where it belongs, standing up for herself. Gaining knowledge about violence and methods to process the experience of violence. Finding her competence in doing new things; changing her surroundings, going to school, finding a new job, new hobbies, etc. Acknowledging the triggers of bad memories and learning how to deal with them. Using her own approaches in dealing with her traumatic experiences; prayer and belief in a higher power, doing things that make her happy, relaxing, and fooling around, not drinking alcohol, processing her childhood experiences, etc.
The Perpetrator	Minimizing the communication with the perpetrator as much as possible, setting boundaries (i.e., restraining order, divorce), not minding his opinion, not defending him.
Children and Loved Ones	Asking her children for forgiveness, forgiving others. Cherishing her loved ones, setting the boundaries needed to be in control of her own life.
Other People	Choosing constructive people and situations in her life. Seeking help in the right places, consulting with people with similar experience. Experiencing love from others and not being judged by others. Ending social isolation. Not taking things too personally. Attention from the other sex gave some of the women confidence.

**Table 5:** Environmental factors facilitating PTG following IPV.

Environmental Factors	
Informal Support	The women’s closest family, relatives, friends, children, new spouse, and pets provided them with informal support. Their manager at work, colleagues, job, and neighbors could also be sources of informal support.
Systematic Support	The public support system: the health care system, social services, the police, rehabilitation pensions, etc., provided them with systematic support.
Organized Supporting Resources	Psychological interviews, organized special resources and peer support, trauma processing (CPT, HAM, EMDR, etc.), vocational rehabilitation, 12-step work (Al Anon, Coda, Bible School), courses processing, e.g., social anxiety, self-empowerment, etc. One participant talked about her lawyer being a “buffer“ between her and the perpetrator.

**Table 6:** Participants’ perceptions of PTG following IPV as described by majority of the women.

Perceptions of Post-Traumatic Growth		
Aspect	Description	Quotes from Participants
Strength	Experience enormous personal strength. Even if the hindrances pile up in their lives they know that they can conquer. Not afraid anymore.	“*I can do so much more than I thought I could. If you are willing to work on your life, even though it takes time and can be really scary and tough... at the end you will get what you worked for*” (Kathy).
Self-Respect	Respect themselves. Enjoy their own company. Proud of themselves.	“*I really enjoy my own company and my soul is peaceful most of the time. I have learned how important it is to take good care of myself. I’m really proud of myself*” (Ursula).
Appreciation	Accept themselves as they are.	“*At last I love my self... I think I deserve all the best*” (Norma).
Boundaries	Set boundaries for themselves and others. Are only responsible for themselves, not others.	“*It has become clear to me, how I want things to be, what I accept from other people and what I do not accept*” (Norma).
Tolerance	More tolerant toward others. More humble. Kind. Patient. Understanding.	“*I have learned to be humble towards other people and to respect their experience*” (Harriet).
Awareness	Have learned a lot about themselves and know themselves better	“*I have learned so much about myself. Who I am, how I feel, what I want, what I need and so on. I’ve also learned what I don’t want in my life*” (Ursula).
Independence	More independent and brave.	“*I can make a big decision and make it happen, I don’t need permission from anyone. I can do everything I want to, I’m an independent and good person*” (Beth).
Selfhood	Difficult experience that made them who they are. They are whole.	“*This experience made me the woman that I am today... I could be drinking and whining every day but I choose not to. I figured things out instead and became so strong and happy... I became me*” (Paula).
Happiness	They are happy and enjoy life. Feel good most days.	“*I’m so happy, I feel really good. Even though I feel sad some days... I know the best ways to handle those feelings and work on my welfare*” (Mona).
Nurture	Take better care of themselves. Work on their happiness. Love themselves.	“*I know I have to take good care of myself and I do. I have this routine, that I need to feel good, the more I take care of myself, the better I feel*” (Lorna).
Vision	The future is bright.	“*The future... there is so much I can do... I really look forward to it*” (Olivia).
Helpfulness	Want to use their experience to help others.	“*I really want to use my experience to help other people who have suffered violence... that’s why I am participating in this study*” (Rose).
Resilience	Difficult experience that resulted in positive things.	“*To be in a violent relationship is a terrible experience. Even so, the growth that followed is so enormous, so precious... I wouldn’t want to be without that growth*” (Joan).
Empowerment	Feel in control of their lives.	“*I have learned so much. I feel so empowered. I’m in control of my life... that is the things that are controllable. I know I will get through everything. I will never give up*” (Ingrid).
Reinforcement	Seek help when needed. Important to get the right help.	“*To seek help when you need it...in places where you know you will get the help and support that you need...that was a very important lesson to learn*” (Ursula).
Determination	Know what they want for themselves in intimate relationships. Focused on what they want and what they need. Fight for justice.	“*If I will find another partner in the future one thing is clear: I’m not going to fix you, I’m not going to be your mother, I want a spouse that is my equal*” (Georgia).

## Data Availability

The data presented in this study are with the corresponding author. Because of anonymity and ethical and personal reasons, the data are not available.
